# Geographic Disparities in Oral Health Among Schoolchildren in Damascus, Syria, During the Prolonged Crisis: A Cross-Sectional Study

**DOI:** 10.7759/cureus.107932

**Published:** 2026-04-29

**Authors:** Alaa Ashour, Mayssoon Dashash

**Affiliations:** 1 Pediatric Dentistry, Damascus University, Damascus, SYR

**Keywords:** geographic disparities, gingivitis, oral health, syrian crisis, dental caries

## Abstract

Background

The prolonged humanitarian crisis in Syria has severely impacted healthcare infrastructure and nutritional security, yet the geographic distribution of oral health outcomes among schoolchildren remains poorly understood.

Objectives

This study aimed to assess geographic disparities in the prevalence of dental caries and gingivitis among children aged six to 12 years in Damascus and to identify district-level variations in oral health status.

Methods

A cross-sectional study was conducted on 872 schoolchildren selected via multistage cluster sampling from nine administrative districts of urban Damascus. Clinical examinations assessed dental caries using DMFT (Decayed, Missing, and Filled Teeth (permanent dentition)) and dmft (decayed, missing, and filled teeth (primary dentition)) indices, gingival health using the Gingival Index (GI), and oral hygiene using the Plaque Index (PI). Data on sociodemographic factors and oral hygiene behaviors were collected via parental questionnaires. Chi-square tests and analysis of variance (ANOVA) were used to compare outcomes across districts, with statistical significance set at p<0.05.

Results

The overall prevalence of dental caries was 733 (84%), and gingivitis was 760 (87%). Significant geographic disparities were observed for the prevalence of dental caries. The highest mean DMFT was in Al‑Mazzeh district (2.62±1.67), while the lowest was in Al‑Qanawat district (1.61±1.44). For primary teeth (dmft), the highest mean was in Al‑Muhajireen district (3.50±2.64) and the lowest in Al‑Qusour district (2.29±2.07). Caries prevalence ranged from 71 (77%) in Al‑Qadam to 84 (90.3%) in Al‑Muhajireen. No statistically significant differences in gingivitis severity were observed across districts (p=0.566). Public schools consistently had worse oral health outcomes than private schools (p=0.01 for caries prevalence; p=0.035 for mean DMFT).

Conclusion

Significant geographic disparities in oral health exist among Damascus schoolchildren, with disadvantaged districts and public schools bearing the highest burden. These findings highlight the urgent need for targeted, area-specific preventive interventions in crisis-affected settings.

## Introduction

Oral diseases, particularly dental caries and gingivitis, remain major public health challenges among children worldwide, with prevalence disproportionately high in low- and middle-income countries [1,2]. In Syria, the ongoing humanitarian crisis since 2011 has devastated the healthcare system, displaced millions, and exacerbated food insecurity, all of which directly impact child oral health [3,4]. Previous Syrian studies have reported high caries prevalence ranging from 61% to 91% [5-7], but most have aggregated data across entire cities without examining intra-urban geographic variations.

Understanding the geographic distribution of disease is not merely an academic exercise; it is an essential tool for public health planning. In stable settings, spatial disparities in oral health are typically attributed to factors such as access to dental clinics, water fluoridation, population density, and income levels [8,9]. However, in conflict settings like Syria, these disparities can be magnified due to uneven destruction of infrastructure, population displacement patterns, unequal distribution of humanitarian aid, and interruption of basic municipal services (e.g., waste collection, water supply) in some areas more than others [10,11].

Although previous studies in Damascus have reported high caries rates (ranging from 61% to 91%) [5-7], all of them aggregated data at the city level or used convenience samples from limited schools without true geographic representation. No previous study has systematically compared oral health outcomes across the different administrative districts of Damascus. This represents a critical knowledge gap, because without knowing which districts are most in need, scarce health resources (e.g., school-based awareness teams, subsidized toothpaste, mobile dental clinics) are distributed randomly or based on accessibility rather than actual need [12].

Furthermore, school type (public vs. private) may serve as an important geographic and social proxy for oral health disparities. In the Syrian context, private schools typically cater to families with higher income and education levels, who can afford better nutrition, oral hygiene products, and dental care. Public schools, which serve the vast majority of children, often lack resources for oral health programs. However, no previous study has examined whether school-type disparities persist after adjusting for geographic location.

This study, therefore, aimed to assess the prevalence and severity of dental caries and gingivitis among schoolchildren aged six to 12 years across all nine districts of Damascus, identify districts with the highest oral disease burden, and compare oral health outcomes between public and private schools as a secondary geographic-social indicator. Unlike previous Syrian studies, this is the first to produce a preliminary "map" of oral health disparities within Damascus city during the prolonged crisis.

## Materials and methods

Study design, setting, and ethical considerations

This cross-sectional study was conducted between September 2023 and May 2024 in urban Damascus, Syrian Arab Republic. The study followed the STROBE (Strengthening the Reporting of Observational Studies in Epidemiology) guidelines and was approved by the Scientific Research Ethics Committee of the University of Damascus (approval no: UDDS/373; date: February 28, 2023) and the Syrian Ministry of Education. Written informed consent was obtained from parents/guardians. All procedures complied with the Declaration of Helsinki.

Sample size calculation

The sample size was calculated using Cochran's formula for prevalence studies. Assuming a 95% confidence level (Z=1.96), a caries prevalence of 79.1% (p=0.791) from a Syrian study in a similar age group [5], and a margin of error of ε=0.03 to increase precision for geographic subgroup analyses, the minimum required sample was 691. To account for the design effect of cluster sampling (estimated design effect=1.2) and potential non-response, the sample size was increased to 872 children.

Geographic stratification and sampling

Urban Damascus is officially divided into nine administrative districts: Al-Qanawat, Al-Qusour, Al-Maliki, Al-Muhajireen, Dumar, Al-Qadam, Al-Mazzeh, Kafr Soussa, and Al-Midan. These districts vary markedly in population density, average income, housing quality, and infrastructure stability during the crisis, ranging from relatively affluent areas (e.g., Al-Mazzeh, Kafr Soussa) to lower-income, high-density areas (e.g., Al-Muhajireen, Al-Qadam).

A multistage cluster sampling strategy was employed to ensure representativeness across all districts:

Stage 1 (District level): All nine districts were included with a predetermined allocation of schools proportional to the number of elementary schools in each district (probability proportional to size (PPS)). The target was three schools per district.

Stage 2 (School level): From each district, three elementary schools were randomly selected from the official list provided by the Damascus Education Directorate using a simple random number generator. If a selected school declined, it was replaced by the next school on the list.

Stage 3 (Class and child level): Within each selected school, three classes were chosen randomly (one from lower, middle, and upper elementary grades to capture the full age range six to 12 years). All children in the selected classes who met the eligibility criteria were invited to participate.

Eligibility criteria

Inclusion criteria were Syrian nationality, age six to 12 years, lifelong residence in Damascus (to avoid confounding by differential exposure to crisis conditions), and parental consent. Exclusion criteria were fixed orthodontic appliances, developmental enamel defects, any diagnosed chronic systemic disease (diabetes, heart disease, immunodeficiency), mental or physical disabilities preventing examination, current smoking, or uncooperative behavior during the clinical examination.

Data collection tools

Parental Questionnaire

A structured, pretested Arabic-language questionnaire was completed by parents/guardians. The questionnaire collected sociodemographic and geographic data: child's age, gender, school name (used to assign district), and school type (public/private). The questionnaire was developed by the authors based on relevant literature and was pretested for clarity. It did not include any copyrighted or proprietary scales. The full questionnaire is provided in the Appendices.

Clinical Oral Examination

All examinations were performed by a single calibrated examiner (AA) in a mobile dental unit set up in each school, under natural daylight supplemented by a headlamp (light emitting diode (LED), 5500 lux). Standard infection control measures included sterile mouth mirrors, World Health Organization Community Periodontal Index (WHO CPI) probes, disposable gloves, and surgical masks.

The following were collected:

Dental caries was recorded using the dmft (decayed, missing, and filled teeth (primary teeth)) and DMFT (Decayed, Missing, and Filled Teeth (permanent dentition)) indices according to World Health Organization criteria [13]. Only cavitated lesions or surfaces with clear undermined enamel or dentinal shadowing were scored as carious. Non-cavitated white spot lesions were recorded as sound.

Gingivitis was assessed using the Gingival Index (GI) developed by Löe and Silness (1963) [14] on six index teeth (or their substitutes). The mean GI score was categorized as mild (0.1-1.0), moderate (1.1-2.0), or severe (2.1-3.0).

Oral hygiene was assessed using the Simplified Oral Hygiene Index (OHI-S) developed by Greene and Vermillion [15], which combines debris and calculus scores. OHI-S was categorized as good (0.0-1.2), fair (1.3-3.0), or poor (3.1-6.0).

Before the main study, the examiner (AA) underwent a training on 30 children not included in the final sample. Intra-examiner reliability was assessed by re-examining 10% of the sample (n=87) after two weeks. Kappa coefficients were 0.89 for caries diagnosis and 0.85 for gingival scoring, indicating excellent agreement.

The indices used in this study (DMFT/dmft, GI, and OHI-S) are standardized epidemiological tools widely used in oral health research and do not require special permission for use. All indices were applied according to their original published protocols.

Data analysis

Data were entered into IBM SPSS, version 25 (IBM Corp., Armonk, NY, USA). Descriptive statistics (frequencies, percentages, means, and standard deviations) were calculated for all variables. Chi-square tests were used to compare categorical outcomes (caries prevalence, gingivitis severity categories) across the nine districts. One-way analysis of variance (ANOVA) was used for continuous variables. The assumption of homogeneity of variances was checked using Levene's test; if violated, Welch's ANOVA was used. Independent t-tests (for mean DMFT) and Chi-square tests (for prevalence and gingivitis severity) were used to compare public vs. private schools. All tests were two-tailed, with a significance level set at p<0.05.

## Results

A total of 872 children (49% men, 51% women; mean age 9.1±1.8 years) participated. Majority of the children were from public school (605, 69.4%) and the rest from private schools (267, 30.6%). Detailed sample distribution by district is shown in Table 1.

**Table 1:** Distribution of Study Sample by District and School Type.

District	Total n (%)	Public Schools n (%)	Private Schools n (%)
Al-Qanawat	109 (12.5)	78 (71.6)	31 (28.4)
Al-Qusour	85 (9.7)	60 (70.6)	25 (29.4)
Al-Maliki	84 (9.6)	59 (70.2)	25 (29.8)
Al-Muhajireen	93 (10.7)	65 (69.9)	28 (30.1)
Dumar	95 (10.9)	66 (69.5)	29 (30.5)
Al-Qadam	92 (10.5)	64 (69.6)	28 (30.4)
Al-Mazzeh	117 (13.5)	81 (69.2)	36 (30.8)
Kafr Soussa	109 (12.5)	76 (69.7)	33 (30.3)
Al-Midan	88 (10.1)	56 (63.6)	32 (36.4)
Total	872 (100)	605 (69.4)	267 (30.6)

**Figure 1:**
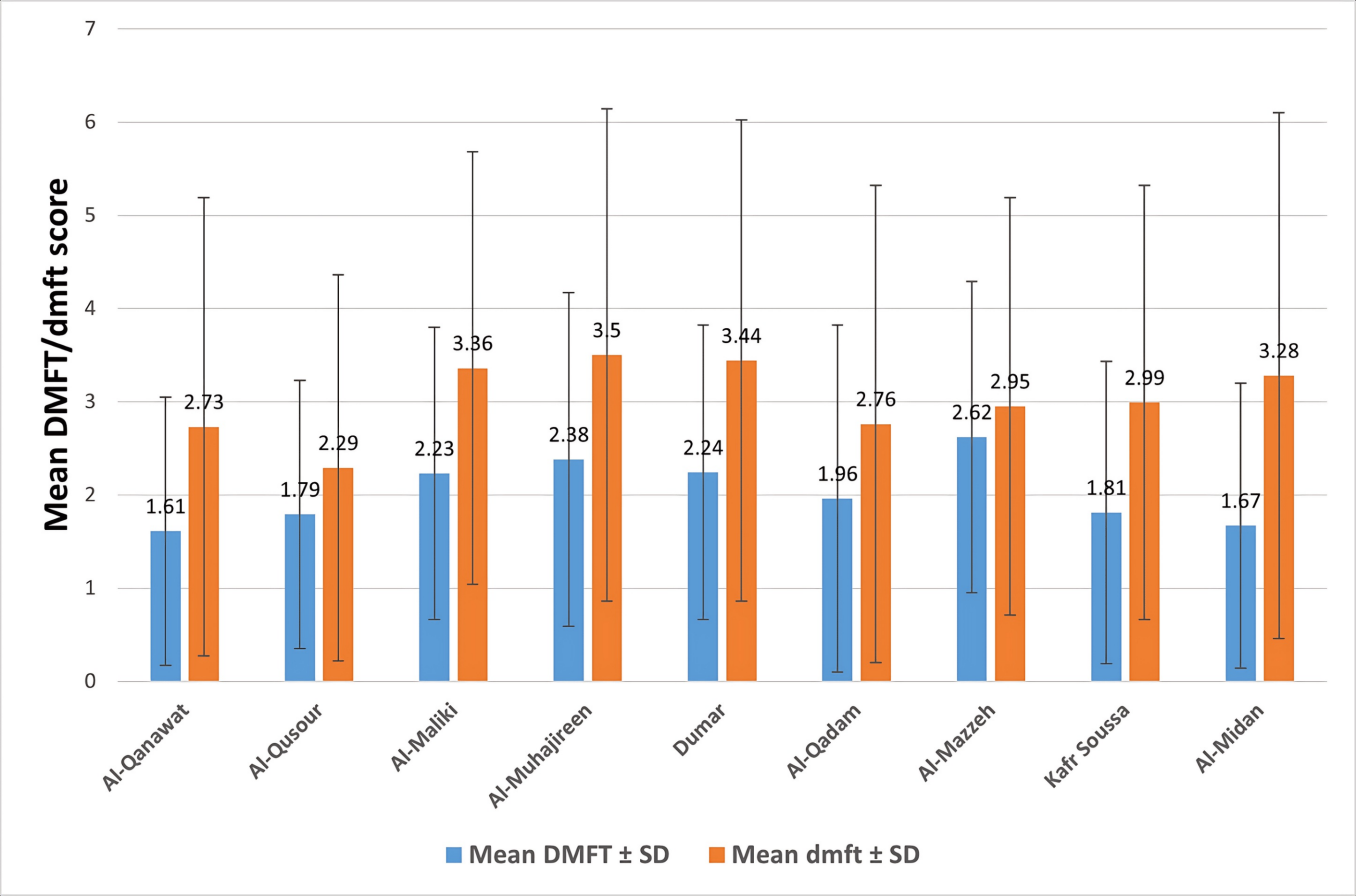
The mean DMFT and dmft scores across nine districts with standard deviation (SD) error bars. Al-Mazzeh has the highest DMFT; Al-Muhajireen has the highest dmft DMFT=Decayed, Missing, Filled permanent teeth; dmft=decayed, missing, filled primary teeth.

Overall caries prevalence was 733 (84%), with significant variation across districts (p=0.010; Table 2). The highest prevalence was in Al-Muhajireen (84, 90%) and the lowest in Al-Qadam (71, 77.2%). For permanent teeth (DMFT), the mean score ranged from 1.61±1.44 in Al-Qanawat to 2.62±1.67 in Al-Mazzeh (p<0.001; Table 3 and Figure 1). For primary teeth (dmft), the mean ranged from 2.29±2.07 in Al-Qusour to 3.50±2.64 in Al-Muhajireen (p=0.016; Table 3).

**Table 2:** Caries Prevalence by District. *Statistically significant at p<0.05; Chi-square test was used.

District	Total N	Caries Present, n (%)	Caries Absent, n (%)	χ² value	p-value
Al-Qanawat	109	88 (80.7)	21 (19.3)	20.1	0.010^*^
Al-Qusour	85	68 (80.0)	17 (20.0)		
Al-Maliki	84	74 (88.1)	10 (11.9)		
Al-Muhajireen	93	84 (90.3)	9 (9.7)		
Dumar	95	84 (88.4)	11 (11.6)		
Al-Qadam	92	71 (77.2)	21 (22.8)		
Al-Mazzeh	117	103 (88.0)	14 (12.0)		
Kafr Soussa	109	96 (88.1)	13 (11.9)		
Al-Midan	88	74 (84.1)	14 (15.9)		

**Table 3:** Mean DMFT and dmft Scores by District. *Statistically significant at p<0.05; One-way analysis of variance (ANOVA) test was used. DMFT=Decayed, Missing, Filled permanent teeth; dmft=decayed, missing, filled primary teeth.

District	N	Mean DMFT±SD	Mean dmft±SD
Al-Qanawat	109	1.61±1.44	2.73±2.46
Al-Qusour	85	1.79±1.44	2.29±2.07
Al-Maliki	84	2.23±1.57	3.36±2.32
Al-Muhajireen	93	2.38±1.79	3.50±2.64
Dumar	95	2.24±1.58	3.44±2.58
Al-Qadam	92	1.96±1.86	2.76±2.56
Al-Mazzeh	117	2.62±1.67	2.95±2.24
Kafr Soussa	109	1.81±1.62	2.99±2.33
Al-Midan	88	1.67±1.53	3.28±2.82
F value		4.85	2.35
p-value		<0.001^*^	0.016^*^

Overall, the prevalence of gingivitis was 87% (760). Among children with gingivitis (GI>0), it was mild in 434 (57%), moderate in 288 (38%), and severe in 38 (5%). Although district-level differences did not reach statistical significance for GI (p=0.566), Al-Muhajireen had the highest proportion of moderate/severe gingivitis (47, 57%) and Al-Midan the lowest (29, 40%) (Table 4).

**Table 4:** Gingivitis Severity Among Children with Gingivitis (GI>0) by District Chi-square test was used; Percentages are calculated among children with gingivitis only.; Children with healthy gingiva (GI=0) are not included in this table.

District	Mild, n (%)	Moderate, n (%)	Severe, n (%)	χ² value	p-value
Al-Qanawat	51 (54.3)	40 (42.6)	3 (3.2)	13.2	0.566
Al-Qusour	47 (62.7)	25 (33.3)	3 (4.0)		
Al-Maliki	47 (63.5)	23 (31.1)	4 (5.4)		
Al-Muhajireen	36 (43.4)	41 (49.4)	6 (7.2)		
Dumar	45 (56.3)	31 (38.8)	4 (5.0)		
Al-Qadam	44 (52.4)	35 (41.7)	5 (6.0)		
Al-Mazzeh	57 (55.9)	38 (37.3)	7 (6.9)		
Kafr Soussa	63 (66.3)	29 (30.5)	3 (3.2)		
Al-Midan	44 (60.3)	26 (35.6)	3 (4.1)		

The mean Plaque Index (PI) score for the study population was 1.43±0.75, indicating a generally moderate level of plaque accumulation. Mean PI scores showed slight variation across districts, with higher values observed in Al-Muhajireen and Al-Qadam, and lower values in Al-Qanawat and Kafr Soussa. However, these differences were not statistically significant (p=0.125; Table 5).

**Table 5:** Mean Plaque Index (PI) by District. One-way analysis of variance (ANOVA) test was used.

District	n	Mean±SD	F value	p-value
Al-Qanawat	109	1.32±0.48	1.72	0.125
Al-Qusour	85	1.41±0.50		
Al-Maliki	84	1.45±0.53		
Al-Muhajireen	93	1.61±0.55		
Dumar	95	1.44±0.51		
Al-Qadam	92	1.52±0.54		
Al-Mazzeh	117	1.46±0.52		
Kafr Soussa	109	1.34±0.49		
Al-Midan	88	1.47±0.56		

The distribution of oral hygiene status, as measured by OHI-S, showed slight variations across districts. The highest proportion of 'good' oral hygiene was in Al-Qanawat (38, 34.9%), and the lowest was in Al-Muhajireen (22, 23.7%). However, these geographic differences were not statistically significant (p>0.05; Table 6).

**Table 6:** Distribution of OHI-S by District. Chi-square test was used.

District	Good, n (%)	Fair, n (%)	Poor, n (%)	χ² value	p-value
Al-Qanawat	38 (34.9)	54 (49.5)	17 (15.6)	18.5	0.245
Al-Qusour	25 (29.4)	44 (51.8)	16 (18.8)		
Al-Maliki	24 (28.6)	44 (52.4)	16 (19.0)		
Al-Muhajireen	22 (23.7)	46 (49.5)	25 (26.9)		
Dumar	26 (27.4)	49 (51.6)	20 (21.1)		
Al-Qadam	24 (26.1)	48 (52.2)	20 (21.7)		
Al-Mazzeh	33 (28.2)	62 (53.0)	22 (18.8)		
Kafr Soussa	37 (33.9)	54 (49.5)	18 (16.5)		
Al-Midan	25 (28.4)	45 (51.1)	18 (20.5)		

Children in public schools had significantly higher caries prevalence (86.3% vs. 79.4%; p=0.010) and higher mean DMFT (2.11±1.63 vs. 1.88±1.67, p=0.035) compared to private schools. No significant differences were found for dmft, PI, or severity of gingivitis (Table 7).

**Table 7:** Comparison of Oral Health Outcomes between Public and Private Schoolchildren in Damascus. *Statistically significant at p<0.05; Independent t-test and chi-square test were used as appropriate. DMFT=Decayed, Missing, Filled permanent teeth; dmft=decayed, missing, filled primary teeth.

Outcome	Public Schools (n=605)	Private Schools (n=267)	Test statistic	p-value
Caries Prevalence (%)	86.3%	79.4%	χ²=6.6	0.010^*^
Mean DMFT (±SD)	2.11±1.63	1.88±1.67	t=2.11	0.035^*^
Mean dmft (±SD)	3.07±2.46	2.96±2.49	t=0.59	0.557
Mean Plaque Index (±SD)	1.45±0.75	1.40±0.75	t=0.70	0.48
Gingivitis Severity (% mod/severe)	43.5%	40.4%	χ²=0.32	0.570

## Discussion

The findings of this study reveal significant intra-urban variations in oral health among schoolchildren in Damascus, with certain districts (Al-Muhajireen, Al-Mazzeh, Al-Maliki) bearing a disproportionately high burden of dental caries and gingivitis, while others (Al-Qanawat, Al-Qusour, Al-Midan) fared relatively better. These disparities persisted despite the overall high prevalence rates.

The highest DMFT score was observed in Al-Mazzeh (2.62), a district with a mixed socioeconomic profile that includes some of the most affluent neighborhoods in Damascus. This seemingly paradoxical finding may be explained by higher sugar consumption in more affluent areas due to greater availability of processed foods and sweets, a pattern documented in dietary studies from the Eastern Mediterranean region during economic instability [16].

Conversely, Al-Muhajireen, a lower-income district characterized by high population density and a substantial number of internally displaced families living in crowded housing, had the highest dmft (3.50) and the highest proportion of moderate/severe gingivitis.

These findings align with Yousaf et al. and Kumar et al. studies, which show that low socioeconomic status and housing instability are strong predictors of poor oral health, particularly in primary teeth [17,18]. The disruption of daily routines and limited kitchen facilities in such settings often lead to increased consumption of cheap, cariogenic snacks and reduced supervision of oral hygiene practices.

The lowest caries prevalence was observed in Al-Qusour (80%) and Al-Qanawat (80.7%). These centrally located, historic districts have traditionally housed many governmental and service facilities. It is plausible that these areas experienced slightly more consistent municipal services - such as waste collection and water supply - and maintained better access to remaining functional public dental clinics during the crisis compared to peripheral or densely populated informal settlements. However, it is crucial to note that even these "better" districts exhibit prevalence rates far exceeding WHO targets, which aim for less than 20% caries prevalence in 12-year-olds [19].

A particularly noteworthy finding is the discrepancy between caries outcomes and hygiene indicators when comparing school types. Children in public schools had significantly higher caries prevalence and severity (DMFT) than their private school counterparts, a trend consistent with studies from other regions [20,21]. However, no significant differences were observed between the two groups regarding mean PI, OHI-S distribution, or gingivitis severity. This divergence suggests that during a prolonged humanitarian crisis, fundamental hygiene practices - specifically, the frequency and quality of toothbrushing - may be universally compromised across socioeconomic strata due to shared hardships such as water shortages, psychological stress, and the unaffordability of basic hygiene products. Nevertheless, the significantly higher DMFT scores in public schoolchildren indicate that children from lower-income families experience more severe consequences of this plaque accumulation. This is likely attributable to a lack of access to restorative dental care and protective measures such as professional fluoride varnish application or pit-and-fissure sealants, services that remain accessible to families with greater financial means who enroll their children in private schools [22]. This disparity underscores the need for targeted preventive interventions within the public school system.

Potential confounding factors should also be considered when interpreting these findings. Socioeconomic variation within districts, differences in household income, parental education, access to dental services, dietary habits, fluoride exposure, and displacement status may have influenced oral health outcomes. Although school type was used as a socioeconomic proxy, it may not fully capture individual-level socioeconomic differences within each district.

Studies from other conflict-affected regions, including Iraq, Palestine, and Libya, have similarly reported high caries prevalence with significant geographic variations [23,24]. However, most of these studies did not map intra-urban differences at the district level. Our study adds valuable granularity by identifying specific high-priority districts within Damascus, thereby enabling more precise allocation of scarce health resources.

Limitations

This study has several limitations. The cross-sectional design precludes causal inference regarding the relationship between geographic location and oral health outcomes. The study did not directly assess individual fluoride exposure or detailed access to dental services. Furthermore, the study was limited to urban Damascus and may not generalize to rural areas or internally displaced populations living in camp settings. Despite these limitations, the study provides valuable evidence on geographic disparities in oral health among schoolchildren in Damascus. These limitations do not undermine the overall findings but should be considered when interpreting the results. Future studies using longitudinal designs and more detailed socioeconomic, behavioral, and environmental variables are recommended to further clarify the observed associations.

## Conclusions

Significant geographic disparities in dental caries exist among schoolchildren in Damascus, with high-density districts and public schools bearing the highest burden. No significant geographic differences in gingivitis severity were identified. These findings underscore the urgent need for geographically targeted, school-based oral health interventions - such as supervised toothbrushing programs and fluoride varnish applications - as a critical component of the humanitarian response in crisis-affected Syria. Future research should employ longitudinal designs to assess the long-term impact of the crisis on oral health trajectories and evaluate the effectiveness of area-level preventive interventions.

## Appendices

**Table 8:** Parental Questionnaire.

Section	Variable	Question	Response Options
Demographic	Age	What is your child’s age?	___ years
Demographic	Gender	What is your child’s gender?	Male / Female
Geographic	School Name	What is the name of your child’s school?	Open-ended
Geographic	District	(Derived from school location)	Al-Qanawat / Al-Qusour / Al-Maliki / Al-Muhajireen / Dumar / Al-Qadam / Al-Mazzeh / Kafr Soussa / Al-Midan
Socioeconomic Proxy	School Type	What type of school does your child attend?	Public / Private
